# Supplementary material for: Modeling glioblastoma heterogeneity as a dynamic network of cell states
Source: Mol Syst Biol. 2021 Sep 16;17(9):e10105. doi: 10.15252/msb.202010105 (PMC8444284; doi:10.15252/msb.202010105)
Supplement: Supplementary file 6 — Source Data for Figure 5 [file MSB-17-e10105-s004.zip › Figure5A_sourcedata/GSEA_3071/nosigdb_stateIII.GseaPreranked.1622795155562/RICHARDS_ZHONG_NATURE_2018_ASTROCYTES_UPREG.html]

Details for gene set RICHARDS\_ZHONG\_NATURE\_2018\_ASTROCYTES\_UPREG[GSEA]

|  || Dataset | state33071 |
| Phenotype | NoPhenotypeAvailable |
| Upregulated in class | na\_neg |
| GeneSet | RICHARDS\_ZHONG\_NATURE\_2018\_ASTROCYTES\_UPREG |
| Enrichment Score (ES) | -0.45650923 |
| Normalized Enrichment Score (NES) | -2.9705167 |
| Nominal p-value | 0.0 |
| FDR q-value | 0.0 |
| FWER p-Value | 0.0 |
Table: GSEA Results Summary

  

Fig 1: Enrichment plot: RICHARDS\_ZHONG\_NATURE\_2018\_ASTROCYTES\_UPREG      
 Profile of the Running ES Score & Positions of GeneSet Members on the Rank Ordered List

  

| PROBE | GENE SYMBOL | GENE\_TITLE | RANK IN GENE LIST | RANK METRIC SCORE | RUNNING ES | CORE ENRICHMENT || 1 | HSPB1 |  |  | 4 | 0.650 | 0.0087 | No |
| 2 | MT2A |  |  | 31 | 0.499 | 0.0093 | No |
| 3 | RHOC |  |  | 92 | 0.414 | -0.0003 | No |
| 4 | GNG5 |  |  | 139 | 0.382 | -0.0067 | No |
| 5 | LGALS1 |  |  | 230 | 0.323 | -0.0256 | No |
| 6 | EFHD2 |  |  | 383 | 0.266 | -0.0617 | No |
| 7 | CTSL |  |  | 492 | -0.254 | -0.0864 | No |
| 8 | TMEM132B |  |  | 578 | -0.258 | -0.1049 | No |
| 9 | LITAF |  |  | 582 | -0.259 | -0.1018 | No |
| 10 | MAGT1 |  |  | 611 | -0.260 | -0.1053 | No |
| 11 | PPP1R16A |  |  | 648 | -0.262 | -0.1109 | No |
| 12 | ZFP36L2 |  |  | 671 | -0.263 | -0.1127 | No |
| 13 | EEPD1 |  |  | 685 | -0.264 | -0.1122 | No |
| 14 | PBXIP1 |  |  | 771 | -0.268 | -0.1306 | No |
| 15 | PLAGL1 |  |  | 783 | -0.268 | -0.1295 | No |
| 16 | MFGE8 |  |  | 794 | -0.269 | -0.1281 | No |
| 17 | ETV5 |  |  | 818 | -0.270 | -0.1301 | No |
| 18 | LRRTM3 |  |  | 827 | -0.271 | -0.1282 | No |
| 19 | THBS3 |  |  | 835 | -0.271 | -0.1260 | No |
| 20 | BCHE |  |  | 867 | -0.272 | -0.1301 | No |
| 21 | CRYL1 |  |  | 939 | -0.276 | -0.1447 | No |
| 22 | PMP22 |  |  | 1023 | -0.281 | -0.1623 | No |
| 23 | PHYHIPL |  |  | 1087 | -0.284 | -0.1747 | No |
| 24 | TFPI |  |  | 1141 | -0.287 | -0.1844 | No |
| 25 | SPATA6 |  |  | 1225 | -0.291 | -0.2019 | No |
| 26 | METTL7A |  |  | 1280 | -0.294 | -0.2117 | No |
| 27 | BBS2 |  |  | 1404 | -0.300 | -0.2396 | No |
| 28 | FADS2 |  |  | 1437 | -0.302 | -0.2435 | No |
| 29 | LRP4 |  |  | 1454 | -0.303 | -0.2432 | No |
| 30 | ETV1 |  |  | 1495 | -0.306 | -0.2492 | No |
| 31 | ROBO3 |  |  | 1527 | -0.308 | -0.2528 | No |
| 32 | OLFM2 |  |  | 1548 | -0.309 | -0.2534 | No |
| 33 | SPRY2 |  |  | 1592 | -0.311 | -0.2601 | No |
| 34 | DAG1 |  |  | 1598 | -0.312 | -0.2567 | No |
| 35 | ALDOC |  |  | 1613 | -0.313 | -0.2557 | No |
| 36 | GPX3 |  |  | 1638 | -0.315 | -0.2574 | No |
| 37 | SELENBP1 |  |  | 1655 | -0.315 | -0.2569 | No |
| 38 | C3orf70 |  |  | 1698 | -0.318 | -0.2632 | No |
| 39 | SLITRK2 |  |  | 1725 | -0.320 | -0.2652 | No |
| 40 | SCD |  |  | 1737 | -0.321 | -0.2633 | No |
| 41 | NT5E |  |  | 1769 | -0.323 | -0.2667 | No |
| 42 | ID2 |  |  | 1780 | -0.323 | -0.2645 | No |
| 43 | CLDN10 |  |  | 1822 | -0.325 | -0.2704 | No |
| 44 | FAM84B |  |  | 1975 | -0.334 | -0.3055 | No |
| 45 | ANOS1 |  |  | 2002 | -0.335 | -0.3073 | No |
| 46 | ARNTL |  |  | 2021 | -0.337 | -0.3070 | No |
| 47 | PLEKHB1 |  |  | 2032 | -0.337 | -0.3046 | No |
| 48 | TIMP1 |  |  | 2038 | -0.337 | -0.3009 | No |
| 49 | HMGCS1 |  |  | 2060 | -0.339 | -0.3014 | No |
| 50 | SIRPA |  |  | 2072 | -0.339 | -0.2992 | No |
| 51 | TRIB2 |  |  | 2105 | -0.342 | -0.3025 | No |
| 52 | LGALS3BP |  |  | 2152 | -0.345 | -0.3095 | No |
| 53 | TNFRSF19 |  |  | 2165 | -0.346 | -0.3074 | No |
| 54 | SASH1 |  |  | 2231 | -0.351 | -0.3193 | No |
| 55 | PAQR8 |  |  | 2246 | -0.352 | -0.3177 | No |
| 56 | GJA1 |  |  | 2362 | -0.362 | -0.3426 | No |
| 57 | TTYH1 |  |  | 2397 | -0.365 | -0.3461 | No |
| 58 | PRCP |  |  | 2489 | -0.372 | -0.3645 | No |
| 59 | ABAT |  |  | 2521 | -0.375 | -0.3671 | No |
| 60 | HEY1 |  |  | 2593 | -0.381 | -0.3801 | No |
| 61 | DHCR7 |  |  | 2654 | -0.386 | -0.3901 | No |
| 62 | ID1 |  |  | 2685 | -0.389 | -0.3922 | No |
| 63 | NOTCH3 |  |  | 2759 | -0.395 | -0.4055 | No |
| 64 | ABHD3 |  |  | 2805 | -0.400 | -0.4114 | No |
| 65 | SEMA6D |  |  | 2812 | -0.400 | -0.4070 | No |
| 66 | CYP51A1 |  |  | 2826 | -0.402 | -0.4044 | No |
| 67 | ALCAM |  |  | 2853 | -0.403 | -0.4052 | No |
| 68 | ELOVL2 |  |  | 2879 | -0.406 | -0.4058 | No |
| 69 | AASS |  |  | 2929 | -0.412 | -0.4125 | No |
| 70 | JUN |  |  | 2935 | -0.413 | -0.4077 | No |
| 71 | ITGAV |  |  | 2942 | -0.414 | -0.4031 | No |
| 72 | MRC2 |  |  | 3032 | -0.424 | -0.4202 | No |
| 73 | SEMA6A |  |  | 3042 | -0.425 | -0.4162 | No |
| 74 | SLC1A4 |  |  | 3051 | -0.426 | -0.4119 | No |
| 75 | SLC3A2 |  |  | 3063 | -0.427 | -0.4084 | No |
| 76 | GPRC5B |  |  | 3092 | -0.430 | -0.4094 | No |
| 77 | SPON1 |  |  | 3187 | -0.441 | -0.4275 | No |
| 78 | MYO10 |  |  | 3196 | -0.442 | -0.4230 | No |
| 79 | MSMO1 |  |  | 3241 | -0.450 | -0.4279 | No |
| 80 | HTRA1 |  |  | 3242 | -0.450 | -0.4212 | No |
| 81 | CD63 |  |  | 3258 | -0.452 | -0.4184 | No |
| 82 | ARHGEF26 |  |  | 3304 | -0.461 | -0.4233 | No |
| 83 | PLEC |  |  | 3324 | -0.466 | -0.4214 | No |
| 84 | DOK5 |  |  | 3428 | -0.487 | -0.4412 | No |
| 85 | ZFP36L1 |  |  | 3487 | -0.503 | -0.4490 | Yes |
| 86 | TRIL |  |  | 3505 | -0.510 | -0.4458 | Yes |
| 87 | DIO2 |  |  | 3510 | -0.511 | -0.4393 | Yes |
| 88 | SLC1A3 |  |  | 3516 | -0.513 | -0.4329 | Yes |
| 89 | TSPAN7 |  |  | 3528 | -0.520 | -0.4280 | Yes |
| 90 | TM7SF2 |  |  | 3558 | -0.530 | -0.4277 | Yes |
| 91 | SOX9 |  |  | 3560 | -0.531 | -0.4200 | Yes |
| 92 | CD82 |  |  | 3569 | -0.534 | -0.4142 | Yes |
| 93 | SERPINE2 |  |  | 3573 | -0.537 | -0.4069 | Yes |
| 94 | LAMB2 |  |  | 3588 | -0.542 | -0.4025 | Yes |
| 95 | LRRC17 |  |  | 3590 | -0.543 | -0.3947 | Yes |
| 96 | SPRY1 |  |  | 3605 | -0.552 | -0.3901 | Yes |
| 97 | SOX2 |  |  | 3613 | -0.555 | -0.3836 | Yes |
| 98 | CD9 |  |  | 3634 | -0.567 | -0.3804 | Yes |
| 99 | MLC1 |  |  | 3659 | -0.584 | -0.3780 | Yes |
| 100 | PNPLA3 |  |  | 3667 | -0.589 | -0.3711 | Yes |
| 101 | PDPN |  |  | 3676 | -0.597 | -0.3642 | Yes |
| 102 | PDGFRB |  |  | 3678 | -0.598 | -0.3556 | Yes |
| 103 | FAT1 |  |  | 3685 | -0.604 | -0.3481 | Yes |
| 104 | CA12 |  |  | 3694 | -0.611 | -0.3411 | Yes |
| 105 | BMP7 |  |  | 3698 | -0.613 | -0.3327 | Yes |
| 106 | GNG7 |  |  | 3722 | -0.633 | -0.3293 | Yes |
| 107 | DCN |  |  | 3723 | -0.633 | -0.3198 | Yes |
| 108 | AIF1L |  |  | 3725 | -0.635 | -0.3106 | Yes |
| 109 | MEIS1 |  |  | 3727 | -0.637 | -0.3013 | Yes |
| 110 | STON2 |  |  | 3739 | -0.658 | -0.2944 | Yes |
| 111 | KLHDC8A |  |  | 3750 | -0.672 | -0.2870 | Yes |
| 112 | TIMP3 |  |  | 3755 | -0.677 | -0.2779 | Yes |
| 113 | ITPR2 |  |  | 3758 | -0.682 | -0.2682 | Yes |
| 114 | FAM181B |  |  | 3760 | -0.686 | -0.2582 | Yes |
| 115 | F3 |  |  | 3764 | -0.693 | -0.2486 | Yes |
| 116 | SOX8 |  |  | 3797 | -0.737 | -0.2461 | Yes |
| 117 | NCAN |  |  | 3813 | -0.761 | -0.2386 | Yes |
| 118 | ELN |  |  | 3818 | -0.766 | -0.2282 | Yes |
| 119 | WSCD1 |  |  | 3835 | -0.798 | -0.2205 | Yes |
| 120 | CLU |  |  | 3846 | -0.827 | -0.2108 | Yes |
| 121 | S100B |  |  | 3853 | -0.845 | -0.1997 | Yes |
| 122 | LRRN3 |  |  | 3859 | -0.856 | -0.1882 | Yes |
| 123 | FABP7 |  |  | 3867 | -0.888 | -0.1768 | Yes |
| 124 | SCRG1 |  |  | 3869 | -0.904 | -0.1635 | Yes |
| 125 | TNC |  |  | 3874 | -0.951 | -0.1504 | Yes |
| 126 | SLC1A2 |  |  | 3877 | -0.969 | -0.1364 | Yes |
| 127 | C1orf61 |  |  | 3879 | -0.986 | -0.1219 | Yes |
| 128 | PTN |  |  | 3884 | -1.022 | -0.1077 | Yes |
| 129 | NPNT |  |  | 3886 | -1.025 | -0.0926 | Yes |
| 130 | GPM6B |  |  | 3889 | -1.047 | -0.0775 | Yes |
| 131 | APOE |  |  | 3891 | -1.056 | -0.0620 | Yes |
| 132 | CXCL14 |  |  | 3892 | -1.060 | -0.0461 | Yes |
| 133 | ID4 |  |  | 3893 | -1.061 | -0.0302 | Yes |
| 134 | GFAP |  |  | 3907 | -1.280 | -0.0145 | Yes |
| 135 | PTPRZ1 |  |  | 3913 | -1.342 | 0.0042 | Yes |
Table: GSEA details [plain text format]

  

Fig 2: RICHARDS\_ZHONG\_NATURE\_2018\_ASTROCYTES\_UPREG: Random ES distribution      
 Gene set null distribution of ES for **RICHARDS\_ZHONG\_NATURE\_2018\_ASTROCYTES\_UPREG**
